# 卤代烷烃脱卤素酶标签*α*_1A_-肾上腺素受体色谱方法的建立与评价

**DOI:** 10.3724/SP.J.1123.2024.04026

**Published:** 2024-10-08

**Authors:** Xinyi YUAN, Liangxi LI, Qin ZHAO, Xiaoying ZHANG, Qian LI, Xinfeng ZHAO, Xu JI

**Affiliations:** 1.西藏民族大学西藏藏医药研究中心藏药活性成分及药理机制研究联合实验室, 陕西 咸阳 712082; 1. Joint Laboratory for Research on Active Components and Pharmacological Mechanism of Tibetan MateriaMedica of Tibetan Medical Research Center of Tibet, Xizang Minzu University, Xianyang 712082, China; 2.西藏民族大学藏药检测技术教育部工程研究中心, 陕西 咸阳 712082; 2. Engineering Research Center of Tibetan Medicine Detection Technology, Ministry of Education, Xizang Minzu University, Xianyang 712082, China; 3.西北大学生命科学学院, 陕西 西安 710069; 3. College of Life Science, Northwest University, Xi’an 710069, China

**Keywords:** *α*_1A_-肾上腺素受体, 卤代烷烃脱卤素酶, 一步固定化, 非线性色谱法, *α*_1A_-adrenergic receptor (*α*_1A_-AR), haloalkane dehalogenase (Halo), one-step immobilization, nonlinear chromatography

## Abstract

受体色谱技术是将色谱技术的高分离能力和药物-受体间的高特异性识别能力结合起来的一种分析技术,能够对中药等复杂体系中靶向活性成分进行高效筛选与准确辨识。该技术的关键在于发展高效、温和、简便的固定化方法,使得固定化受体活性得以保持。传统的以生物交联剂为核心的随意共价固定化技术存在反应特异性较差、需要纯化蛋白质等不足。针对该问题,本研究将卤代烷烃脱卤素酶(Halo)与6-氯己酸的特异性生物正交反应引入至*α*_1A_-肾上腺素受体(*α*_1A_-AR)的固定化过程,将Halo标签*α*_1A_-AR一步固定至6-氯己酸修饰的氨丙基硅胶表面,无需对*α*_1A_-AR进行纯化。采用扫描电子显微镜和色谱法对Halo-*α*_1A_-AR色谱固定相进行形貌及活性表征,证明受体已成功固定且具有特异性识别配体的能力,30天内稳定性良好。非线性色谱法研究结果显示:盐酸哌唑嗪、盐酸特拉唑嗪和乌拉地尔通过一类结合位点与Halo-*α*_1A_-AR色谱固定相作用,结合常数分别为3.85×10^5^、5.00×10^5^和5.90×10^5^ L/mol;甲磺酸酚妥拉明和盐酸坦索罗辛在Halo-*α*_1A_-AR色谱固定相上则存在两类位点,前者与受体亲和力分别为3.12×10^6^ L/mol和6.01×10^5^ L/mol,后者则为9.98×10^5^ L/mol和2.11×10^4^ L/mol。与传统物理吸附法或*N*,*N'*-羰基二咪唑法制备的*α*_1A_-AR色谱柱相比,本文所用固定化方法无需纯化受体,在一定程度上避免了受体活性损失,实现了蛋白质一步固定化方法,亲和力测定值更接近于溶液中受体-药物的真值,为复杂体系靶向活性成分高效筛选与准确测定奠定了基础。

## Abstract (en)

Receptor chromatography is an efficient analytical technique that combines the high separation ability of chromatography with the high specificity of receptors for drug recognition. In addition, this technique offers the advantages of active recognition, online separation, and convenient multidimensional target tracking. This strategy allows target active ingredients in complex systems, such as traditional Chinese medicines, to be efficiently screened and accurately identified. Furthermore, the interactions between ligands and immobilized proteins can be studied. To avoid a loss in function, receptor chromatography requires efficient, mild, and simple immobilization methods that do not damage the structure of the immobilized receptors. Improvements in the activity, stability, and ligand-recognition specificity of immobilized functional proteins can be achieved by selecting appropriate immobilization conditions. Notably, the protein immobilization method is not only closely related to the recognition ability of receptor chromatography but also determines the accuracy of the technique.

Common methods for immobilizing functional proteins include physical adsorption, chemical reactions, biological affinity reactions, and click chemistry. Despite being easy to operate under mild reaction conditions, these methods have shortcomings, including poor reaction specificity and the necessity of using high-purity functional proteins to prepare chromatography columns. Maintaining the high activity of immobilized receptors and ensuring excellent identification and separation abilities are key challenges in the further development of receptor chromatography. In this work, these issues were addressed by introducing a specific bioorthogonal reaction involving haloalkane dehalogenase (Halo) and 6-chlorohexanoic acid for the immobilization of the *α*_1A_-adrenergic receptor (*α*_1A_-AR). Specifically, Halo-*α*_1A_-AR was immobilized on the surface of 6-chlorohexanoic acid-modified aminopropyl silica gel in one step.

The stationary phase with immobilized Halo-*α*_1A_-AR was characterized using scanning electron microscopy. Moreover, the activity of the Halo-*α*_1A_-AR chromatographic column was evaluated using specific ligands (terazosin hydrochloride, phentolamine mesylate, tamsulosin hydrochloride, and urapidil) and nonspecific ligands (yohimbe and metoprolol) for *α*_1A_-AR. Halo-*α*_1A_-AR was successfully immobilized on the silica gel surface with good stability over 30 days, and the Halo-*α*_1A_-AR chromatographic column exhibited good ligand-recognition activity. The nonlinear chromatography results indicated that prazosin hydrochloride, terazosin hydrochloride, and urapidil interacted with immobilized Halo-*α*_1A_-AR through one type of binding site, with association constants of 3.85×10^5^, 5.00×10^5^, and 5.90×10^5^L/mol, respectively. In contrast, phentolamine mesylate and tamsulosin hydrochloride interacted with immobilized Halo-*α*_1A_-AR through two types of binding site. The association constants with the high- and low-affinity binding sites were 3.12×10^6^ and 6.01×10^5^L/mol, respectively, for phentolamine mesylate and 9.98×10^5^ and 0.21×10^5^L/mol, respectively, for tamsulosin hydrochloride.

Compared with the traditional carbonyldiimidazole method, the immobilization method developed in this work did not require receptor purification and thus minimized the loss of receptor activity. The affinity constants obtained with immobilized Halo-*α*_1A_-AR were consistent with the values determined for receptor-ligand binding in solution, indicating that the Halo-*α*_1A_-AR chromatography column is suitable for studying drug-protein interactions. This approach also provides a foundation for the efficient screening and accurate determination of target active ingredients in complex systems.

以药-靶特异性识别过程为核心的受体色谱法是新兴的中药亲和分析研究新方法,能有效解决传统中药学分析方法特异性较差、效率较低等问题。作者前期研究发现,药物在体内和受体作用的过程与色谱系统溶质在固定相上的吸附及解离行为相似,通过将该特点与传统色谱技术高分离能力等优点相结合,提出了将体内药物作用的最大靶点家族蛋白G-蛋白偶联受体(GPCRs)固载于色谱填料表面,建立了受体色谱新方法^[1]^。该方法不仅延续了色谱技术的高分离能力,还凸显了药物识别的靶向性特征,具有活性识别、在线分离、便于多维靶点组合等特点^[2,3]^。据此,课题组先后发展了包含*β*_2_-肾上腺素受体色谱在内的近20种受体色谱模型,对多个中药单药、复方和DNA编码中药分子库进行了研究,为以中药为源泉的创新药物研发提供了方法学借鉴^[4⇓⇓⇓-8]^。

受体色谱法最为关键的步骤是受体蛋白的固定化,固定化过程中可能会破坏受体天然态结构,从而影响其活性,导致后期测定的可靠性与准确性下降。目前常见的功能蛋白质固定化方法根据作用方式不同主要包括物理吸附法、亲和固定法、随意共价固定化法以及点击化学法^[9⇓⇓⇓⇓-14]^。物理吸附法本质上属于非位点特异性、非共价固定化方法,具有操作简便、蛋白质固载量大的优势,但也存在稳定性较差的问题;亲和固定法是利用受体分子与固体基质表面特异性基团之间的亲和作用进行受体特异性固定的方法,这种方法具有统一受体构象、减少受体活性位点损失等优点,但其产物稳定性仍有待进一步提高;随意共价固定化法及点击化学法虽然对产物的稳定性有一定提升,但仍需要对蛋白质进行纯化以减少杂蛋白的干扰,容易对受体活性造成影响。

本文建立了一种基于卤代烷烃脱卤素酶(haloalkane dehalogenase, Halo)与微球表面修饰的基团(卤代烷烃)间特异性生物正交反应来实现受体蛋白质固定化的方法。该方法可以有效减少蛋白质活性位点的丢失,同时具有良好的产物稳定性。源于紫红球菌脱卤素酶演变而成的特异性蛋白酶Halo由293个氨基酸组成,通过使用无水解催化活性的苯丙氨酸(F)代替组氨酸(H),突变脱卤素酶H272F,可使得卤代烷烃与该酶中Asp106以共价键结合^[3,15]^。利用该反应的高度特异性,将突变的Halo融合至*α*_1A_-肾上腺素受体(*α*_1A_-AR)的非活性末端并诱导其表达,用细胞裂解液直接与卤代烷烃修饰的硅胶混合,即可实现*α*_1A_-AR的一步固定,制备*α*_1A_-AR色谱固定相。与传统*α*_1A_-AR固定化方法相比,该方法无需对蛋白质进行纯化,可以有效避免蛋白质纯化过程中受体活性的降低,因而可以更加准确地测定药物-蛋白质的结合情况,为药物-蛋白质相互作用研究提供更加可靠的新方法。

## 1 理论

非线性色谱法(nonlinear chromatography, NLC)于1944年由Thomas推导获得,并在之后由Wade等引入亲和色谱分析中^[16,17]^。该方法假设色谱柱外效应和扩散效应忽略不计,配体和固定化蛋白的结合和解离动力学速率为影响色谱峰变形和展宽的主要因素。相关参数可通过公式(1)和公式(2)进行计算^[17]^。


(1) $$\begin{array}{c} y=\frac{a_{0}}{a_{3}}\left[1-\exp\left(-\frac{a_{3}}{a_{2}}\right)\right]\\ \left\{\frac{\sqrt{\frac{a_{1}}{x}}I_{1}\left(\frac{2\sqrt{a_{1}x}}{a_{2}}\right)\exp\left(\frac{-x-a_{1}}{a_{2}}\right)}{1-T\left(\frac{a_{1}}{a_{2}},\frac{x}{a_{2}}\right)\left[1-\exp\left(-\frac{a_{3}}{a_{2}}\right)\right]}\right\} \end{array}$$

(2) $$T(u,v)=\exp(-v)\int_{0}^{u}\exp(-t)I_{0}(2\sqrt{vt})dt$$

(3) *a*_2_=1/*k*_d_*t*_0_

(4) *a*_3_=*K*_A_*C*_0_

(5) *k*_a_=*k*_d_*K*_A_

其中*I*_0_()、*I*_1_()为调整贝尔塞函数,*a*_0_为峰面积参数,*a*_1_为保留因子参数,*a*_2_为峰展宽参数,*a*_3_为峰变形参数,*k*_d_(/s)为蛋白质与配体的解离速率常数,*t*_0_(min)为色谱系统死时间,*K*_A_(L/mol)为吸附平衡常数,*C*_0_(mmol/L)为进样浓度,*k*_a_(L/(mol·s))为结合速率常数。

## 2 实验部分

### 2.1 试剂与仪器

亚硝酸钠(20201005)购于天津市天力化学试剂有限公司;硅胶购于中科院兰州化学物理研究所;盐酸坦索罗辛(纯度98%, S40584)、乌拉地尔(纯度98%, B26417)购于上海源叶生物科技有限公司;盐酸特拉唑嗪(纯度98%, B-SR340)购于上海贤鼎生物科技有限公司;甲磺酸酚妥拉明(纯度98%, P123277)购于上海阿拉丁试剂有限公司;盐酸哌唑嗪(纯度99%, P838220)、育亨宾(纯度98%, Y820631)、美托洛尔(纯度98%, M875830)购于上海麦克林生化科技有限公司。

LC 2030 PLUS高效液相色谱仪,配紫外检测器,日本岛津公司;SS-326型高压灭菌锅,日本TOMY公司;JY92-II N型超声波细胞粉碎仪,宁波新芝生物科技股份有限公司;LYNX 4000型离心机,赛默飞世尔科技公司;QUANTA 600F扫描电子显微镜,美国FEI公司;BL2000装柱机,浙江正泰电器股份有限公司。

### 2.2 实验方法

#### 2.2.1 目标蛋白Halo-*α*_1A_-AR的诱导表达

将*Halo*基因融合至*α*_1_*_A_*-*AR*基因的C末端,并通过Gateway克隆在大肠杆菌中构建融合基因的原核表达载体^[18,19]^。首先将*attB1*和*attB2*重组位点的*α*_1_*_A_*-*AR* (Gene ID: P35348)编码DNA片段(生工生物工程(上海)股份有限公司)与具有*attP1*和*attP2*位点的大肠杆菌载体重组,然后向100 ng重组质粒中依次添加2 μL BP Clonase^TM^酶混合物、1 μL 5×BP Clonase^TM^反应物,蒸馏水定容至8 μL,将得到的混合物在25 ℃下反应2.0 h,然后加入1 μL *α*_1_蛋白酶K对BP Clonase^TM^酶进行封闭。随后将编码*α*_1_*_A_*-*AR* DNA片段的初级克隆体转化到含有*attR1*和*attR2*位点的目的载体pReceiver-ccdB-Halo中,从而获得pReceiver-*α*_1A_-AR-Halo。将Halo-*α*_1A_-AR重组质粒转染至*E. Coli* BL21(DE3)感受态细胞中,培养得到Halo-*α*_1A_-AR工程菌株。采用TB培养基对该工程菌进行培养,并加入异丙基-*β*-D-硫代半乳糖苷(IPTG)至终浓度为0.5 mmol/L以诱导蛋白表达,培养温度为20 ℃, 220 r/min振荡培养24 h。待培养结束后,4 ℃、6500 r/min离心10 min,收集菌体。对收集的菌体称重,按每1 g沉淀加入破碎细菌裂解液10 mL的比例悬浮菌体,在冰浴条件下超声破碎细胞(300 W,破碎10 s,间隔15 s,破碎时间为25 min),待破碎完全,4 ℃、7500 r/min离心15 min,收集上清液。

#### 2.2.2 Halo-*α*_1A_-AR色谱固定相的制备

使用*γ*-氨丙基三乙氧基硅烷对已活化的大孔硅胶(直径7 μm)进行修饰得到氨丙基硅胶,随后利用酰化反应将6-氯己酸修饰至氨丙基硅胶表面,将6-氯己酸修饰的氨丙基硅胶与Halo-*α*_1A_-AR菌体裂解液的上清液按1 g∶50 mL的比例混合,室温机械搅拌60 min,用20 mmol/L磷酸缓冲液(PB, pH=7.4)洗涤3次,得到Halo-*α*_1A_-AR色谱固定相。采用湿法装柱法将所制备的Halo-*α*_1A_-AR色谱固定相装填至空色谱柱管(50 mm×4.6 mm)中,装填压力为400 bar, 50 min后取下色谱柱,于4 ℃保存备用。

#### 2.2.3 Halo-*α*_1A_-AR色谱固定相的表征

采用扫描电子显微镜(SEM)分别对空白硅胶、氨丙基硅胶、6-氯己酸修饰的硅胶以及Halo-*α*_1A_-AR修饰的硅胶进行形貌观察,所有样品在观察前均在真空干燥箱中60 ℃干燥过夜。在SEM表征前,分别为样品镀覆导电金膜,从而减少检测时所产生的荷电。

采用高效液相色谱系统对Halo-*α*_1A_-AR色谱固定相的活性进行表征。以*α*_1A_-AR的4种特异性配体盐酸特拉唑嗪、盐酸坦索罗辛、甲磺酸酚妥拉明和乌拉地尔为工具药,20 mmol/L PB(pH 7.4)为流动相,紫外检测波长分别为246、275、278、268 nm,柱温为20 ℃,在0.6 mL/min流速下分别记录保留时间*t*_R_(min),以220 nm处亚硝酸钠的保留时间为系统死时间*t*_0_(min),利用公式(6)计算各配体的保留因子*k*:


(6) $$k=\frac{t_{R}-t_{0}}{t_{0}}$$

为了排除非特异性吸附的可能性,在相同色谱条件下对4种特异性工具药在空白硅胶柱上的保留情况进行测定。此外,在278、275 nm波长处分别研究*α*_2_-AR拮抗剂育亨宾和*β*_1_-AR阻滞剂美托洛尔在Halo-*α*_1A_-AR色谱柱上的保留行为,从而判断Halo-*α*_1A_-AR色谱固定相是否可以特异性识别*α*_1A_-AR的配体。

为了考察Halo-*α*_1A_-AR色谱固定相对配体识别能力的稳定性,持续一个月内每天重复3次进样盐酸特拉唑嗪、盐酸坦索罗辛、甲磺酸酚妥拉明和乌拉地尔4种工具药,观察其在Halo-*α*_1A_-AR色谱柱上的保留时间、峰形和峰面积的变化,并分别计算保留时间的相对标准偏差(RSD)。

#### 2.2.4 非线性色谱法研究5种工具药与Halo-*α*_1A_-AR色谱固定相的相互作用

以20 mmol/L PB(pH=7.4)为流动相,流速为0.6 mL/min,在20 ℃条件下分别进样不同浓度(见表1)的5种工具药,记录每种工具药的保留时间。在软件PeakFit v4.12中采用非线性色谱法对各色谱峰进行拟合处理,即可得到*a*_0_、*a*_1_、*a*_2_和*a*_3_等相关参数,按照公式(3)~(5)分别计算5种工具药与*α*_1A_-AR的相互作用参数。

**表1:** 非线性色谱法测定5种工具药与Halo-*α*_1A_-AR色谱固定相相互作用的检测波长及浓度分配

Ligand	Wavelength/nm	Concentrations/(mmol/L)
Prazosin hydrochloride	254	0.025, 0.05, 0.075, 0.1, 0.25, 0.5, 0.75, 1.0, 1.5, 2.0
Terazosin hydrochloride	246	0.01, 0.025, 0.05, 0.075, 0.1, 0.25, 0.5, 0.75, 1.0, 1.5
Phentolamine mesylate	275	0.025, 0.05, 0.075, 0.1, 0.25, 0.5, 0.75, 1.0, 1.5, 2.0
Tamsulosin hydrochloride	278	0.005, 0.01, 0.025, 0.05, 0.075, 0.1, 0.25, 0.5, 0.75, 1.0, 1.5, 2.0
Urapidil	268	0.025, 0.05, 0.075, 0.1, 0.25, 0.5, 0.75, 1.0, 1.5, 2.0

## 3 结果与讨论

### 3.1 目标蛋白Halo-*α*_1A_-AR的诱导表达

与LB培养基相比,TB培养基营养含量更高,可以在一定程度上增加微生物的生长量,从而增加目的蛋白质的表达量^[20,21]^。采用TB培养基对Halo-*α*_1A_-AR工程菌低温培养24 h,采用十二烷基硫酸钠聚丙烯酰胺凝胶电泳(SDS-PAGE)对Halo-*α*_1A_-AR在大肠杆菌中的表达情况进行测定,结果如图1所示。对该蛋白质的重组序列进行分析,计算可知Halo-*α*_1A_-AR的分子质量约为86.5 kDa。由图1可知,细菌裂解液的上清液在66.2~116.2 kDa范围内有一条明显的目的条带,在裂解液的沉淀中则几乎没有该条带,表明Halo-*α*_1A_-AR已成功表达在大肠杆菌中,且主要存在于裂解液的上清液中。

**图1:**
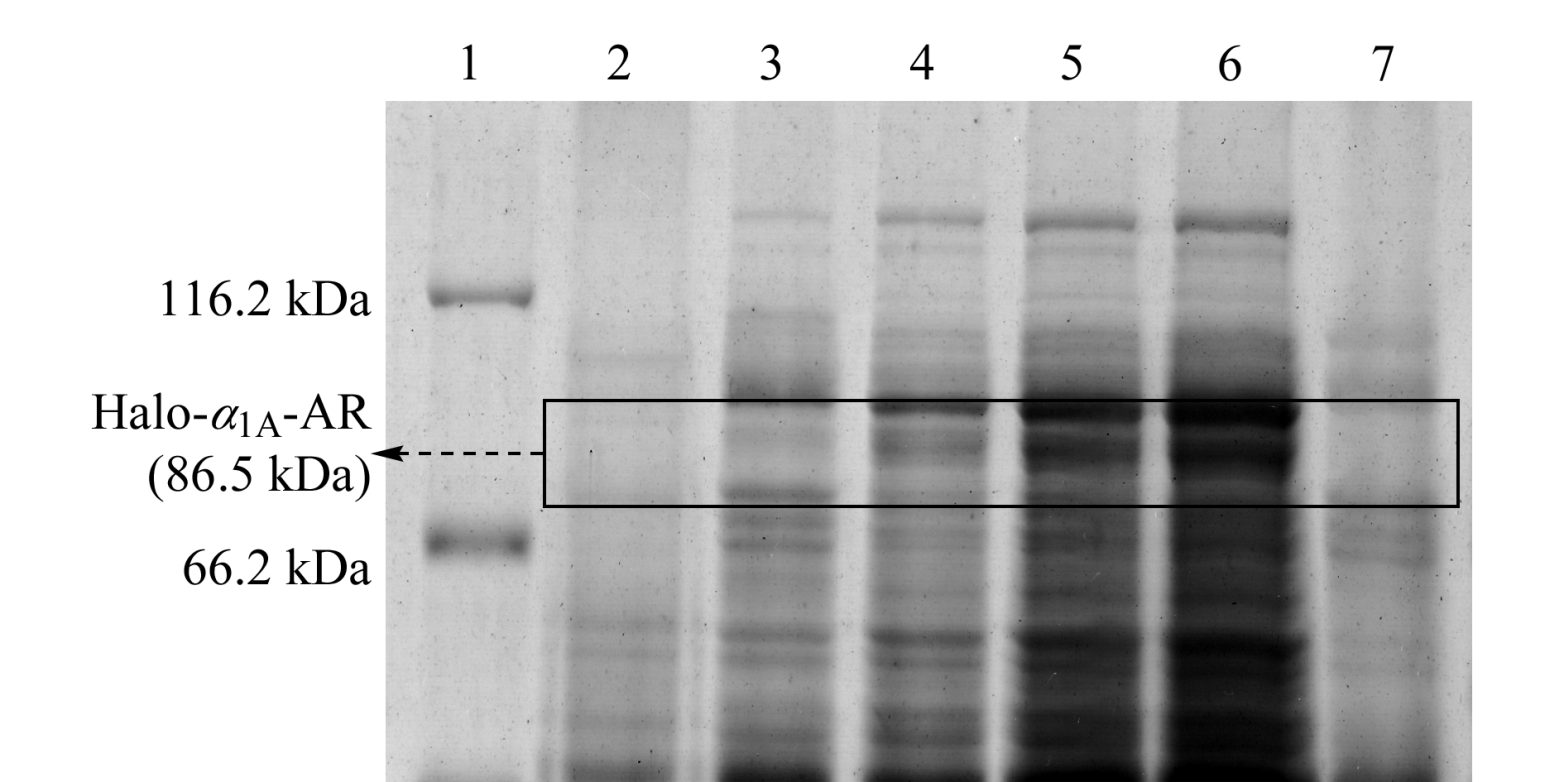
Halo-*α*_1A_-AR表达情况的SDS-PAGE图

### 3.2 Halo-*α*_1A_-AR色谱固定相的表征

#### 3.2.1 形貌表征

将含有Halo-*α*_1A_-AR细胞裂解液的上清液部分与6-氯己酸修饰的氨丙基硅胶直接混合,进行*α*_1A_-AR的定向固定(图2a)。Halo-*α*_1A_-AR色谱固定相制备过程中各步骤硅胶的形貌表征SEM结果如图2b~e所示。由图2b可知,空白硅胶呈球形多孔结构,其直径分布均匀,尺寸为(7.000±0.006) μm。然而,Halo-*α*_1A_-AR修饰的硅胶呈现更粗糙的表面,直径增加到(7.056±0.008) μm(图2e),且表面粗糙程度明显高于前3组硅胶样品(图2b~d),表明Halo-*α*_1A_-AR通过在硅胶微球表面单层分布成功地固定在硅胶表面。为了证明该固定化方法非简单的物理吸附,本研究用不同浓度的磷酸盐缓冲液对固定化受体进行了多次洗涤,与未洗涤的硅胶相比,其形态和直径无明显变化,证实受体与硅胶的结合相对稳定。

**图2:**
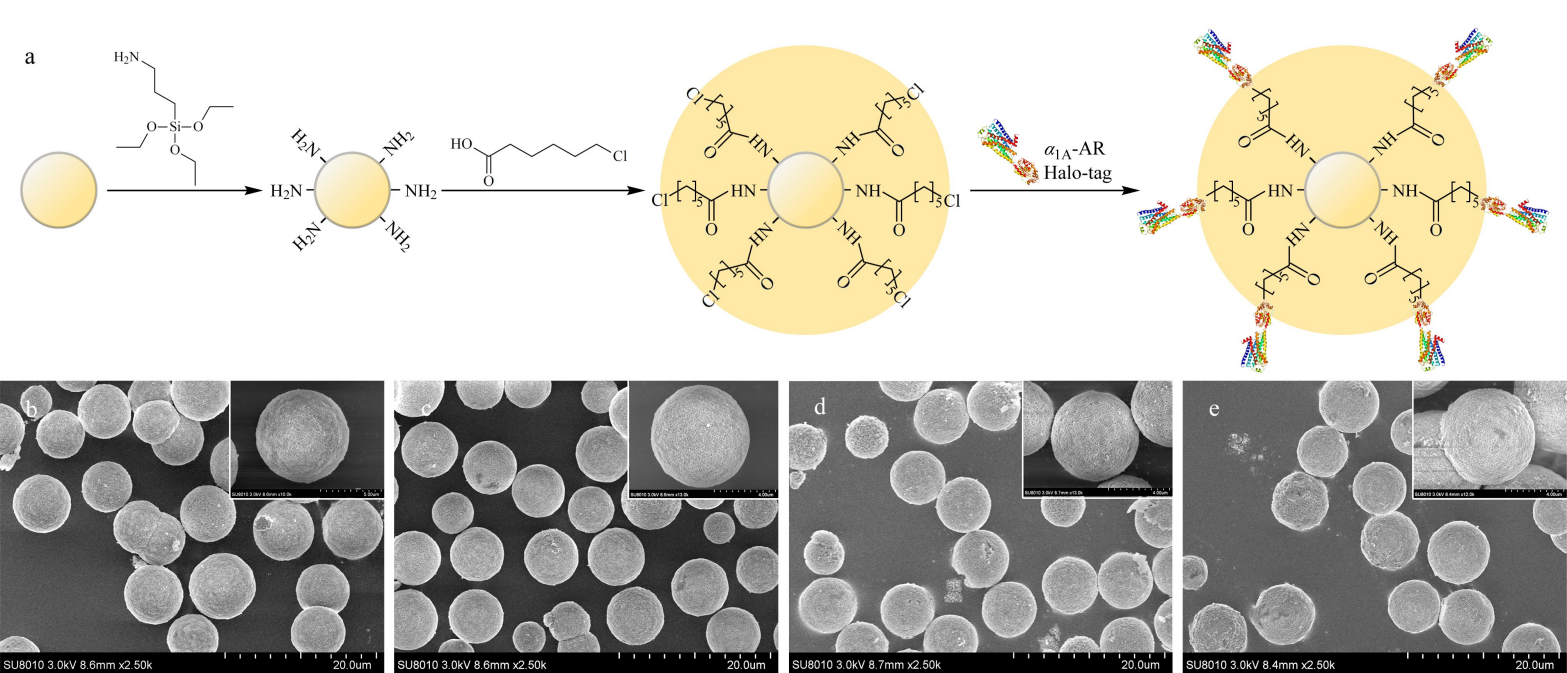
Halo-*α*_1A_-AR色谱固定相的(a)制备及其(b~e)形貌表征

#### 3.2.2 活性表征

通过测定盐酸特拉唑嗪、甲磺酸酚妥拉明、盐酸坦索罗辛和乌拉地尔4种工具药在Halo-*α*_1A_-AR色谱柱上的保留行为对Halo-*α*_1A_-AR色谱固定相的活性进行特异性表征,结果如图3所示。在2.2.3节所述色谱条件下,当流速为0.6 mL/min时,通过亚硝酸钠测得系统死时间为1.04 min,而4种工具药的保留时间分别为11.59、13.31、9.20和5.23 min,均远大于系统死时间(图3a),证明这4种工具药可以被Halo-*α*_1A_-AR色谱固定相识别,计算4种工具药的*k*分别为10.15±0.12、11.79±0.11、7.85±0.09和4.00±0.07。在相同条件下,这4种工具药在空白对照柱上的保留时间依次为1.18、1.22、1.24、1.08 min,接近系统死时间,由此可以排除这4种工具药在Halo-*α*_1A_-AR色谱固定相上非特异性吸附的可能性(图3b)。在相同流速下,测定*α*_2_-AR拮抗剂育亨宾和*β*_1_-AR阻滞剂美托洛尔在Halo-*α*_1A_-AR色谱柱上的保留情况,保留时间分别为1.12和1.28 min,同样接近该流速下的系统死时间,表明二者在Halo-*α*_1A_-AR色谱柱上均无明显保留(图3c),由此证明本论文所构建的Halo-*α*_1A_-AR色谱固定相对工具药的识别具有亚型特异性,可以准确识别到作用于*α*_1A_-AR亚型的工具药。

**图3:**
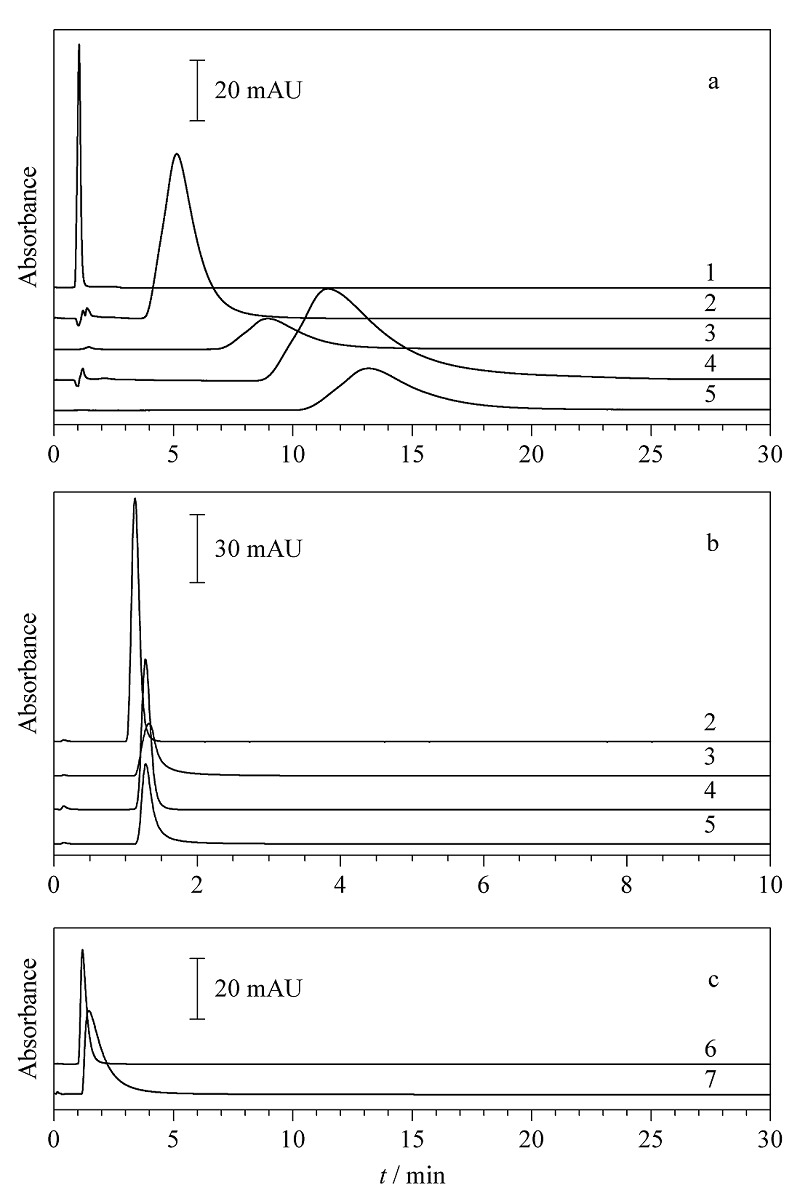
Halo-*α*_1A_-AR色谱固定相的活性表征

#### 3.2.3 稳定性考察

稳定性考察结果表明,4种工具药在Halo-*α*_1A_-AR色谱柱上的保留情况在一个月内无明显改变,盐酸特拉唑嗪、盐酸坦索罗辛、甲磺酸酚妥拉明和乌拉地尔保留时间的RSD值分别为1.7%、1.5%、2.1%、1.9%,证明所构建的Halo-*α*_1A_-AR色谱固定相对药物的识别能力在一个月内无明显变化。

### 3.3 非线性色谱法研究5种工具药与Halo-*α*_1A_-AR色谱固定相的相互作用

由于配体与蛋白质的结合有快吸附慢解离的特点,一定程度上会形成拖尾峰,非线性色谱法则适用于拖尾峰的分析研究。将测得的色谱图数据导出,其中保留时间记为*X*,吸光度记为*Y*,通过色谱系统定量环体积*V*、流速*v*、进样浓度*C*_0_及系统死时间*t*_0_可以计算出*C=C*_0_*V/vt*_0_,分别利用*x=X/t*_0_、*y=Y/C*对*X*、*Y*进行归一化处理。归一化后的数据以修正保留时间为横坐标,归一化吸光度为纵坐标,使用软件PeakFit v4.12对色谱峰基线进行去除,采用Savitzky-Golay算法对色谱峰进行除噪,在不改变色谱峰原始形状的前提下,采用公式(1)和公式(2)对色谱峰进行重新拟合。图4为20 ℃时,0.01 mmol/L特拉唑嗪在Halo-*α*_1A_-AR色谱柱上非线性拟合前后的色谱图(其中深色线条1为拟合前的色谱图,浅色线条2为经非线性拟合后的色谱图)。其余浓度及工具药拟合前后的色谱图见附图S1~S5(详见https://www.chrom-China.com)。经非线性色谱法拟合处理后,得到了*a*_0_、*a*_1_、*a*_2_和*a*_3_等相关参数。

**图4:**
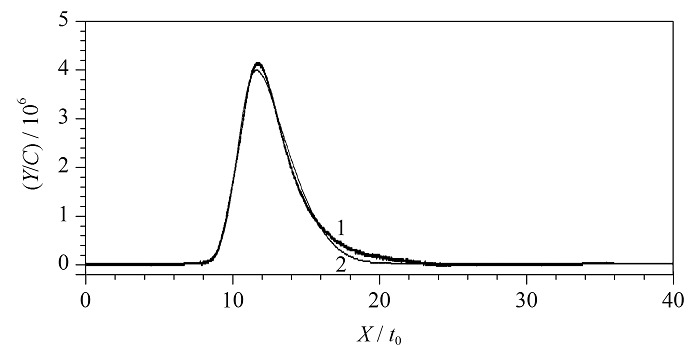
盐酸特拉唑嗪(0.01 mmol/L)非线性拟合前后的色谱图

根据*a*_0_、*a*_1_、*a*_2_和*a*_3_等参数,采用公式(3)~(5),分别计算出相应浓度范围内5种工具药的*k*_d_、*k*_a_和*K*_A_,计算结果见表2。可以看出,盐酸哌唑嗪、盐酸特拉唑嗪、乌拉地尔3种工具药与Halo-*α*_1A_-AR色谱固定相的结合常数*K*_A_分别为3.85×10^5^、5.00×10^5^和5.90×10^5^L/mol;甲磺酸酚妥拉明在低浓度(0.025~0.1 mmol/L)和高浓度(0.25~2.0 mmol/L)时的结合常数*K*_A_分别是3.12×10^6^ L/mol和6.01×10^5^L/mol;盐酸坦索罗辛在低浓度(0.005~0.1 mmol/L)和高浓度(0.25~2.0 mmol/L)时的结合常数*K*_A_分别是9.98×10^5^L/mol和2.11×10^4^L/mol。

**表2:** 非线性色谱法计算5种工具药与Halo-*α*_1A_-AR色谱固定相的相互作用参数

Ligand	C^*^/ (mmol/L)	/ s^-1^	/ (10^6^ L/(mol·s))	K_A_/(10^5^ L/mol)	
				Halo-α_1A_-AR	Previous study
Prazosin hydrochloride	0.025-2.0	3.62	1.10	3.85^*^	/
Terazosin hydrochloride	0.01-1.5	8.13	3.76	5.00^*^	2.91^#^/0.79^※^
Phentolamine mesylate	0.025-0.1	7.95	3.73	31.20^*^	1.32^#^
	0.25-2.0	9.08	3.03	6.01^*^	/
Tamsulosin hydrochloride	0.005-0.1	3.24	2.46	9.98^*^	5.35^#^
	0.25-2.0	1.44	0.29	0.21^*^	/
Urapidil	0.025-2.0	2.40	1.20	5.90^*^	/

* Data obtained by nonlinear chromatography from Halo-*α*_1A_-AR chromatography column in this study; # data obtained from immobilized *α*_1A_-AR chromatography column prepared by physical adsorption method in previous study^[23]^; ※ data obtained from immobilized *α*_1A_-AR chromatography column prepared by *N*,*N'*-carbonyldiimidazole method in previous study^[22]^.

前期研究分别采用物理吸附法和生物交联剂*N*,*N'*-羰基二咪唑对*α*_1A_-AR进行了固定,分别测定了不同药物和*N*-苯基哌嗪衍生物与*α*_1A_-AR色谱固定相的亲和参数^[22,23]^。以特拉唑嗪为例,与前期2个研究结果(*K*_A1_: 2.91×10^5^L/mol, *K*_A2_: 7.90×10^4^L/mol)相比,通过本工作所构建的Halo-*α*_1A_-AR固定相分析得到的盐酸特拉唑嗪与*α*_1A_-AR的亲和力明显提升,其原因在于本方法中固定化方式的改进避免了受体的活性损失,使得*α*_1A_-AR对配体的敏感性增加,提升了亲和力。除此之外,本论文所采用的固定化方法为一步固定化方法,无需对受体进行纯化,避免了因蛋白质纯化造成的受体活性损失。

## 4 结论

本研究以6-氯己酸为底物修饰氨丙基硅胶,利用卤代烷烃脱卤素酶与其特异性底物之间的生物正交反应,成功实现了Halo-*α*_1A_-AR色谱固定相一步化制备,无需对蛋白质进行纯化,从而避免了受体蛋白质活性位点的损失。

利用所制备的Halo-*α*_1A_-AR色谱固定相,采用非线性色谱法研究5种工具药与Halo-*α*_1A_-AR色谱固定相的相互作用参数,将其与前期采用物理吸附法或*N*,*N'*-羰基二咪唑实现*α*_1A_-AR固定化的相关研究结果进行对比,发现Halo-*α*_1A_-AR与其配体展现出更高的亲和力,进一步表明本研究所采用的固定化方法可以有效避免受体蛋白质活性的损失,从而在一定程度上提高与配体的亲和力。因此,利用Halo与卤代烷烃的生物正交反应实现受体蛋白质的一步固定化可以更加准确地进行药物-蛋白质相互作用研究。
